# O24 Malignancy in antiphospholipid syndrome: a catastrophe

**DOI:** 10.1093/rap/rkab067.023

**Published:** 2021-10-19

**Authors:** Salema Khalid, Steven Young Min

**Affiliations:** Queen Alexandra Hospital, Portsmouth, United Kingdom

## Abstract

**Case report - Introduction:**

Antiphospholipid syndrome (APS) is a rare autoimmune disease that can cause venous and arterial thrombosis in virtually any organ. The spectrum of vascular events can range from superficial thrombosis to life-threatening multiple organ thromboses (catastrophic APS or CAPS). CAPS occurs in genetically susceptible individuals in response to a “trigger” such as infection, cancer, trauma, surgery, anticoagulation/immunosuppression withdrawal and SLE flares.

The diagnosis of CAPS can be extremely challenging and is associated with a high morbidity and mortality. Thus, early diagnosis and treatment are critical to prevent the progression of disease and improve the prognosis.

**Case report - Case description:**

We report the case of a 78-year-old gentleman who was diagnosed with systemic lupus erythematosus and antiphospholipid syndrome in 2001 after he presented with a DVT, PE, rash and arthralgia. He had positive anti-cardiolipin antibodies, Rheumatoid Factor, Ro and La antibodies, but negative anti-dsDNA. He had remained stable on warfarin, hydroxychloroquine 400mg and prednisolone 7mg for 17 years. In 2018, hydroxychloroquine was reduced to 200mg OD and steroid taper was started. Unfortunately, he presented to the Emergency Department in July 2020 with a left leg swelling. DVT was confirmed on ultrasound, despite a therapeutic INR of 2.4. He was also noted to have thrombocytopenia. Haematology advised this was in keeping with ITP and started him on 70mg of prednisolone daily. No cause for the DVT was seen on CT. However, it did show subpleural nodules within the right costophrenic angle and a repeat CT in 4 months’ time was advised. INR target was increased to 3.0—4.0 and patient was discharged.

He was re-admitted 4 days later with an acute drop in haemoglobin, raised inflammatory markers and worsening kidney function. CT showed extensive retroperitoneal haematoma. It also revealed a PE as well as colonic distension with gradual tapering to normal calibre, thought to represent pseudo-obstruction.

Rheumatology, haematology, general surgery and ITU were involved in the management. He was started on treatment dose clexane, given intravenous immunoglobulins and supportive blood transfusions. IVC filter was put in.

Unfortunately, he dropped his GCS and an urgent CT brain showed a left posterior fossa mass with a bleed. The case was discussed with neurosurgery and neuroradiology who felt that the top differential for the intracranial lesion was an underlying metastasis – particularly a colonic met. Colonoscopy was advised. However, due to severe frailty and multiple pathologies, the patient was made palliative and was fast-tracked home.

**Case report - Discussion:**

Definite CAPS is defined as thromboses in three or more organs developing in less than a week, microthrombosis in at least one organ and persistent antiphospholipid antibody (aPL) positivity. The diagnosis of probable CAPS requires three out of these four criteria. Although pathological confirmation of microthrombosis is one of the requirements for CAPS, biopsy may not be possible during an acute episode due to severe thrombocytopenia and/or unstable clinical course, as in our case. There is another category called ‘CAPS-like’ disease, where aPL-positive patients do not fulfil the definite or probable CAPS criteria. However, they still represent a significant challenge for physicians and require close monitoring and aggressive treatment.

Initially, we felt that we had triggered probable CAPS or ‘CAPS-like’ disease, by reducing his hydroxychloroquine and steroids. However, he did not improve with high-dose steroids given for his thrombocytopenia. Also, autoimmune screen including anti-dsDNA and complement levels were not significant.

CAPS occurs in 46% of patients with a previous diagnosis of APS, and a precipitating factor is present in half the patients. It is speculated that aPL-related clinical events respond to the two-hit theory: a second hit or trigger is needed to activate the prothrombotic properties of aPL, which is the first hit. In CAPS, the most frequently recognised trigger is infection, followed by cancer. A study showed that 9% of patients with CAPS presented with an underlying malignancy, with haematological malignancies being most common, followed by lung and colon carcinoma. Similarly, Ozguroglue et al. showed an association between high level of anticardiolipin antibody and thromboembolic events in patients with colorectal, breast, ovarian, lung, and pancreatic cancer. Recent studies also suggest an increased prevalence of certain cancers in aPL-positive patients, thereby prompting an extensive search for an occult malignancy in such cases.

**Case report - Key learning points:**

Given the increased prevalence of cancers in aPL-positive patients, this case highlights the need to thoroughly investigate for an occult malignancy as a trigger for APS (classic form or CAPS) with a new episode of thrombosis, despite adequate anticoagulation. While we were focusing on tapering of the immunosuppressive medication as a possible trigger, this episode was most likely triggered by the possible metastatic malignancy – especially given the lag of almost 2 years between reduction in hydroxychloroquine and steroids and development of symptoms.

It is also important to bear in mind, especially in elderly patients, that thrombotic events associated with aPL can be the first manifestation of malignancy. This emphasises the need for continuing research on the association between antiphospholipid syndrome and malignancies.

While the survival rate of patients with CAPS is poor overall, the outcome of patients with CAPS is worse in the presence of malignancy. A study showed that only 40% of CAPS patients with malignancies improved. This may be due to the presence of the malignancy as well as the older age of the patients.

We are looking forward to discussing CAPS at the BSR case-based conference and hope it will shed more light on diagnosis and management of this incredibly challenging condition.

